# RADIOTHERAPY NEEDS BY PORTUGUESE OLDER ADULTS IN 2040—A GLOBOCAN ANALYSIS

**DOI:** 10.1093/geroni/igad104.2428

**Published:** 2023-12-21

**Authors:** Edna Darlene Rodrigues, Paulo Almeida, Escarlata Lopez, Laetitia Teixeira

**Affiliations:** School of Medicine and Biomedical Sciences, University of Porto, Porto, Porto, Portugal; Centro Hospitalar Universitário São João, Porto, Porto, Portugal; GenesisCare Spain, Madrid, Madrid, Spain; School of Medicine and Biomedical Sciences, University of Porto, Porto, Porto, Portugal

## Abstract

In Portugal, cancer is the second leading cause of death, accounting 28,544 deaths in 2018 (around 25% of all deaths). Radiotherapy (RT) is an effective cancer treatment. Around half of all cancer patients will require RT as part of their treatment. The European SocieTy for Radiotherapy and Oncology - Health Economics in Radiation Oncology (ESTRO-HERO) project data states that Portuguese actual utilisation of RT relative to the evidence-based optimal use is between 50-60%. The aim of this study was to project RT use and resources to maintain RT accessibility for Portuguese older adults in 2040 for the most common types of cancers. The authors examined GLOBOCAN data to determine expected incidence of prostate, colon, rectum, lung, breast, and stomach cancers for those aged 70 and over. Results show that in 2040, new cancer cases in older adults are expected to account for around 60% of total cancer cases. Cancer incidence is estimated to increase between 30 to 40% in this population. In accordance with this projection, the authors used the optimal utilisation percentage of RT to calculate the number of RT courses anticipated to be needed for each cancer type and an increase of around 38% (8878 to 12729) is expected by 2040 to address the needs of this population. The ESTRO-HERO guidelines state that each RT machine should treat 400 to 450 patients per year. So, to cover the older patients’ demand, we estimate the need to install 8 to 10 additional RT machines by 2040.

